# All-Trans Retinoic Acid Treatment Is Associated with Prohibitin Expression in Renal Interstitial Fibrosis Rats

**DOI:** 10.3390/ijms13032769

**Published:** 2012-03-01

**Authors:** Tian-Biao Zhou, Yuan-Han Qin, Zheng-Yi Li, Hui-Ling Xu, Yan-Jun Zhao, Feng-Ying Lei

**Affiliations:** Department of Pediatrics, The First Affiliated Hospital of GuangXi Medical University, NanNing 530021, China; E-Mails: a126tianbiao@126.com (T.-B.Z.); zhengyili_gx@163.com (Z.-Y.L.); xuhuiling304@gmail.com (H.-L.X.); yanjunzhao08@126.com (Y.-J.Z.); leifengyinggx@yahoo.cn (F.-Y.L.)

**Keywords:** all-trans retinoic acid, prohibitin, extracellular matrix, cell apoptosis, renal interstitial fibrosis, unilateral ureteral obstruction

## Abstract

This study was performed to investigate the association of prohibitin with renal interstitial fibrosis (RIF) lesion and to explore the association of all-trans retinoic acid (ATRA) treatment with prohibitin expression in RIF rats. Rats were divided into three groups: the sham operation group (SHO), the model group subjected to unilateral ureteral obstruction (UUO), and the model group treated with ATRA (GA). Renal tissues were collected at 14 and 28 days after surgery, and the relevant indicators were detected. In comparison with the SHO group, the RIF index in the UUO group was markedly elevated (*p* < 0.01), and the RIF index in the GA group was alleviated compared with that in the UUO group (*p* < 0.01). Compared with the SHO group, the expression of prohibitin (protein or mRNA) in the UUO group was significantly reduced (each *p* < 0.01). Prohibitin expression in the GA group was markedly increased when compared with that in the UUO (*p* < 0.01). The expression of TGF-β1 (protein and mRNA), protein expressions of Col-IV, fibronectin, α-SMA and cleaved Caspase-3, ROS generation and cell apoptosis index in the UUO group were markedly higher than those in the SHO group (all *p* < 0.01), and their expressions in the GA group were markedly down-regulated compared to those in the UUO group (all *p* < 0.01, respectively). The protein expression of prohibitin was negatively correlated with the RIF index, protein expression of TGF-β1, Col-IV, fibronectin, α-SMA or cleaved Caspase-3, ROS generation and the cell apoptosis index (each *p* < 0.01). In conclusion, lower expression of prohibitin is associated with the RIF, and ATRA treatment is associated with increased prohibitin, which can prevent the progression of RIF.

## 1. Introduction

Renal interstitial fibrosis (RIF) is the final common pathway leading to chronic kidney diseases and end-stage renal disease [1,2], and its effective therapeutic strategy is wanting at present. Unilateral ureteral obstruction (UUO) is a well-characterized model of experimental obstructive nephropathy, culminating in RIF [3,4]. Currently, investigations have found that the reactive oxidative species (ROS) can contribute to the progression of renal disease induced by UUO [5,6].

Prohibitin is a ubiquitous protein with a number of different molecular functions [7] and it is mainly located on the inner mitochondrial membrane and nuclei [8]. It plays a pivotal role in the processes of cell differentiation and apoptosis [9]. The over-expression of prohibitin could protect the mitochondria from ROS-induced injury [10]. The abnormal expression of prohibitin might be associated with RIF progression.

All-trans retinoic acid (ATRA), a natural derivative of vitamin A [11], has been shown to have a variety of functions, including a role as an antioxidant and regulator of cell differentiation and apoptosis [12,13]. Some investigations [14–16] reported that ATRA could play a protective role in some renal diseases, but the detailed mechanism was not clear. Interestingly, ATRA treatment can suppress the generation of ROS [12], and have an anti-oxidative effect [17]. As mentioned above, we drew up a hypothesis that ATRA treatment is associated with the expression of prohibitin. This investigation was performed to explore this hypothesis in RIF rats induced by UUO.

## 2. Results

### 2.1. Renal Morphology

**Figure 1:**
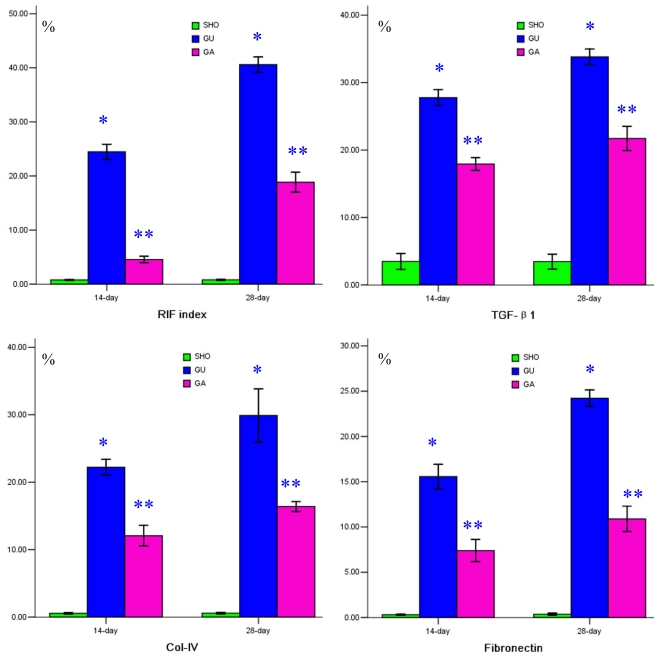

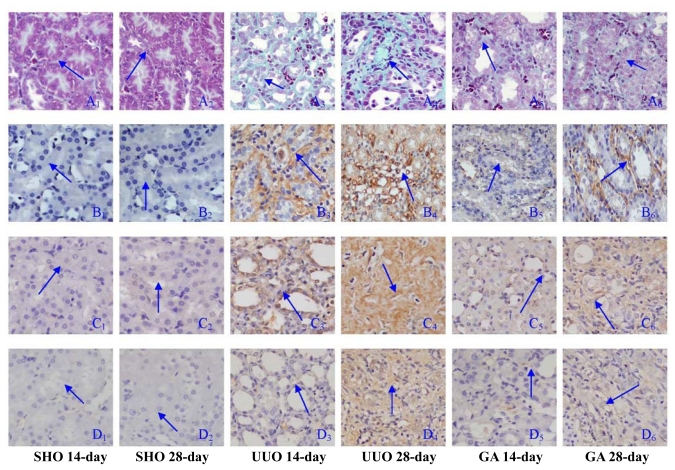
(**1**) Statistical parameters in three groups. *: *p* < 0.01 compared with sham operation group (SHO), **: *p* < 0.01 compared with unilateral ureteral obstruction group (UUO); (**2**) Tissue characteristics in three groups. Masson staining for SHO group (A_1_: 14-day; A_2_: 28-day), UUO group (A_3_: 14-day; A_4_: 28-day) and model group treated with ATRA (GA) (A_5_: 14-day; A_6_: 28-day). Representative samples of immunohistochemical staining for TGF-β1 (SHO: B_1_: 14-day, B_2_: 28-day; UUO: B_3_: 14-day, B_4_: 28-day; GA: B_5_: 14-day; B_6_: 28-day), Col-IV (SHO: C_1_: 14-day, C_2_: 28-day; UUO: C_3_: 14-day, C_4_: 28-day; GA: C_5_: 14-day; C_6_: 28-day), and fibronectin (SHO: D_1_: 14-day, D_2_: 28-day; UUO: D_3_: 14-day; D_4_: 28-day; GA: D_5_: 14-day; D_6_: 28-day) were observed in three groups. Magnification × 400.

Light microscopy showed more collagen deposition, fibroblast proliferation and diffused lymphocytes filtration in the renal tubulointerstitium of the UUO group when compared with those in the SHO group (blue arrowhead in Figure 1), but the lesion degree in the GA group was less serious than that in the UUO group (blue arrowhead in Figure 1). The RIF score in the UUO group was significantly increased over that in the SHO group, and the RIF index in the GA group was markedly alleviated compared with that in the UUO group (all *p* < 0.01; Figure 1).

### 2.2. Association of ATRA Treatment with TGF-βl, Col-IV, Fibronectin, α-SMA, Prohibitin or Cleaved Caspase-3 Protein Expression

**Figure 2:**
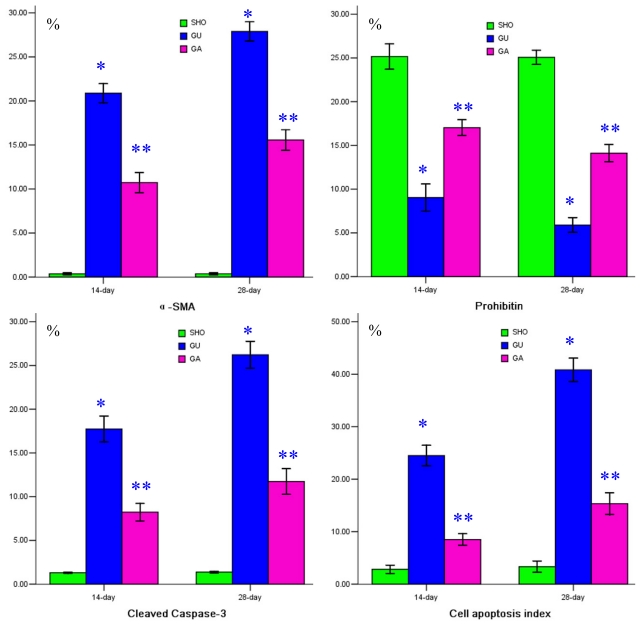

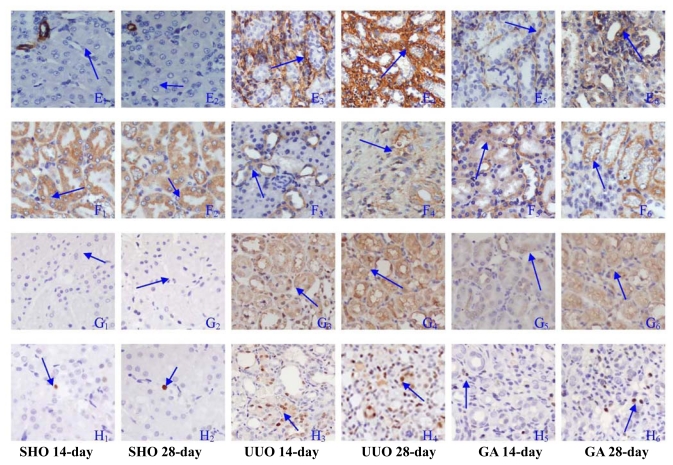
(**1**) Statistical parameters for α-SMA, prohibitin, cleaved Caspase-3 and cell apoptosis index in three groups. *: *p* < 0.01 compared with SHO, **: *p* < 0.01 compared with UUO; (**2**) Tissue characteristics in three groups. Representative samples of immunohistochemical staining for α-SMA (SHO: E_1_: 14-day, E_2_: 28-day; UUO: E_3_: 14-day, E_4_: 28-day; GA: E_5_: 14-day; E_6_: 28-day), prohibitin (SHO: F_1_: 14-day, F_2_: 28-day; UUO: F_3_: 14-day, F_4_: 28-day; GA: F_5_: 14-day; F_6_: 28-day), and cleaved Caspase-3 (SHO: G_1_: 14-day, G_2_: 28-day; UUO: G_3_: 14-day, G_4_: 28-day; GA: G_5_: 14-day; G_6_: 28-day) were observed in three groups. Cell apoptosis also detected in three groups (SHO: H_1_: 14-day, H_2_: 28-day; UUO: H_3_: 14-day, H_4_: 28-day; GA: H_5_: 14-day; H_6_: 28-day). Magnification × 400.

The staining of prohibitin in the SHO group was more significant than that in the UUO group or GA group (blue arrowhead in Figure 2). The stainings of TGF-βl, Col-IV, fibronectin, α-SMA and cleaved Caspase-3 in the UUO group were much more significant compared with those in the SHO group / GA group (blue arrowhead in Figure 1 and 2). Comparison with that in the SHO group, the protein expression of prohibitin in the UUO group was significantly weakened, and protein expression of prohibitin in the ATRA treatment group was markedly increased over that in the UUO group (*p* < 0.01; Figure 2). The protein expressions of TGF-βl, Col-IV, fibronectin, α-SMA and cleaved Caspase-3 in the UUO group were significantly increased when compared with those in the SHO group, and the TGF-βl, Col-IV, fibronectin, α-SMA and cleaved Caspase-3 expressions in the GA group were lower than those in the UUO group (all *p* < 0.01; Figure 1 and 2). Protein expression of prohibitin was negatively correlated with the RIF index, protein expression of TGF-βl, Col-IV, fibronectin, α-SMA or cleaved Caspase-3 (*r* = −0.825, −0.786, − 0.948, −0.817, −0.953, −0.863; each *p* < 0.01, respectively).

### 2.3. Association of ATRA Treatment with Cell Apoptosis

The staining of cell apoptosis of the renal tubulointerstitium was much more significant in the UUO group than in the SHO group (blue arrowhead in Figure 2), and the cell apoptosis index in the UUO group was significantly increased when compared with that in SHO (*p* < 0.01; Figure 2). Cell apoptosis in the renal tubulointerstitium in the ATRA treatment group was attenuated (blue arrowhead in Figure 2), and the apoptosis index in the GA group was significantly reduced over that in the UUO group (*p* < 0.01; Figure 2). Protein expression of prohibitin was negatively correlated with the cell apoptosis index (*r* = −0.886, *p* < 0.01).

### 2.4. Association of ATRA Treatment with ROS Generation, mRNA Expressions of Prohibitin and TGF-βl

**Figure 3:**
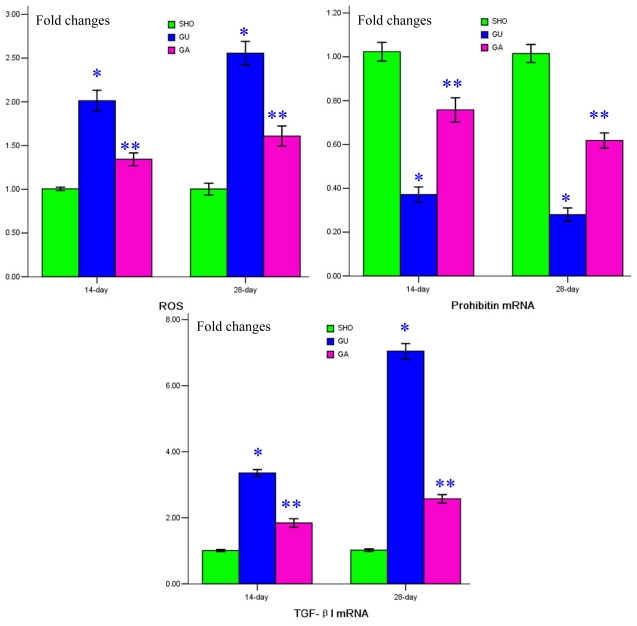
Statistical parameters for reactive oxidative species (ROS), prohibitin mRNA, and TGF-β1 mRNA. *: *p* < 0.01 compared with SHO, **: *p* < 0.01 compared with UUO.

When compared with that in the SHO group, the generation of ROS in the UUO group was markedly increased, especially in 28-days (*p* < 0.01; Figure 3). ROS generation in the GA group was alleviated when compared with that of the UUO group (*p* < 0.01; Figure 3). Protein expression of prohibitin was negatively correlated with ROS generation (*r* = −0.859, *p* < 0.01). Consistently lower prohibitin mRNA expression and higher TGF-βl mRNA expression were shown in the renal tissue of the UUO group when compared with that in the SHO group (all *p* < 0.01; Figure 3). Prohibitin mRNA expression was significantly up-regulated and TGF-βl mRNA expression was markedly reduced in the GA group when compared with the UUO group (all *p* < 0.01; Figure 3).

## 3. Discussion

Of all the cytokines and growth factors involved, TGF-β1 plays a pivotal role in the processes of renal interstitial fibrosis (RIF) [18]. TGF-β1 is known as one of the major mediators that leads to RIF by inducing the production of α-SMA and ECM (Col-IV and fibronectin) in the renal tubulointerstitium [19–21]. Those indicators were chosen and evaluated in this investigation.

Prohibitin is an ubiquitous protein and might play a pivotal role in the process of cell apoptosis, and ATRA treatment can have an antioxidant effect and regulate the cell apoptosis. In this investigation, the ROS generation, the cleaved Caspase-3 and the cell apoptosis index were also evaluated.

In our study, we found that the expressions of TGF-β1, α-SMA, Col-IV and fibronectin in the UUO group were markedly increased when compared to those in the SHO group, especially in 28-days. However, the expression of prohibitin in the UUO group was markedly reduced over that in SHO. Protein expression of prohibitin was negatively correlated with the RIF index, protein expression of TGF-βl, α-SMA, Col-IV, fibronectin or cleaved Caspase-3, ROS generation or cell apoptosis index. The number of apoptotic cells in the UUO group was much more than in the normal control group. The lower expression of prohibitin was associated with increased cell apoptosis and RIF lesion. Interestingly, the prohibitin mainly located in renal tubular epithelial cells (RTEC) and apoptotic cells, observed by us, was mainly contributed by RTEC in our study. We speculated that the mechanism might be as follows: The expression of prohibitin in RTEC in the UUO model was reduced. The generation of TGF-βl and ROS was increased, which induced the expressions of α-SMA, Col-IV and fibronectin in the renal tubulointerstitium and increased the production of cleaved Caspase-3 in RTEC. The cell apoptosis index was also up-regulated. The less expression of prohibitin was associated with increased expressions of ROS, TGF-βl, α-SMA, Col-IV, fibronectin, cleaved Caspase-3 and the index of cell apoptosis.

Prohibitin is a highly conserved, ubiquitously expressed protein that participates in diverse processes including mitochondrial chaperone, growth and apoptosis [22] and is regarded as an apoptosis-regulating protein [23]. Prohibitin has played a protective role in some studies. Theiss *et al*. [24] reported that the elevation of prohibitin in the surface epithelial cells of the colon could reduce the severity of colitis in mice, suggesting that prohibitin might represent a novel therapeutic target in inflammatory bowel disease. Ko *et al*. [22] found that hepatocyte-specific prohibitin deficiency resulted in marked liver injury, oxidative stress, and fibrosis with development of hepatocellular carcinoma, suggesting that the prohibitin was a tumor suppressor in hepatocytes. Muraguchi *et al*. [25] performed an investigation into H9C2 cardiomyocytes, and found that prohibitin might function as a survival factor against hypoxia-induced cell death. Liu *et al*. [10] conducted a study in cardiomyocytes, and their data indicated that prohibitin could protect the cardiomyocytes from oxidative stress-induced damage. The results from these studies mentioned above drew a consistent conclusion that prohibitin was associated with injury of cells or tissues. The results were similar to ours. So, how to regulate the abnormal expression of prohibitin is very important; prohibitin might be a potential therapeutic target for regulation of cell apoptosis and prevention of cell or tissue injury. However, cell culture using RTEC, *etc*., and the inhibition of the signalling pathway of prohibitin need to be conducted to explore further its detailed mechanism in the progression of RIF.

In this investigation, we found that ATRA treatment was associated with increased expression of prohibitin, attenuation of the lesion of the renal tubulointerstitium and reduction in the expressions of TGF-βl, α-SMA, Col-IV, fibronectin, ROS and cleaved Caspase-3. Kishimoto *et al*. [26] also performed an investigation on the association of ATRA treatment with RIF progression in UUO model mice, and found that a significant improvement was observed in histological and immunological findings in the ATRA treatment group. Their results were similar to ours.

There was no report investigating whether ATRA treatment was associated with the expression of prohibitin *in vivo* or *in vitro*. Li *et al*. [9] showed that prohibitin was localized in the nuclear matrix in human neuroblastoma SK-N-SH cells and its distribution was altered due to retinoic acid treatment, but they did not explore whether the retinoic acid treatment was associated with prohibitin expression.

## 4. Materials and Methods

### 4.1. Animal Model

Wistar male rats (6-week-old) were purchased from the Experimental Animal center of Guangxi Medicial University, Nanning, China. The rats were divided into 3 groups: sham operation group (SHO), model group subjected to unilateral ureteral obstruction (UUO), model group treated with ATRA (GA); *n* = 40, respectively. The ureter was ligated at approximately 1 cm below the renal hilum with a 3–0 silk suture. The abdominal wound was closed, and the rats were returned to the cages. Control rats underwent abdominal incision and approximation with no ligation of the ureter [27,28]. The rats in the GA group were treated with ATRA (Sigma Co., USA; 15 mg/kg·d) in corn oil once daily by oral gavage, from the first day before operation. Rats in the UUO and SHO groups were administered with saline. Twenty rats of the three groups were sacrificed 14 and 28 days after surgery respectively, and the renal tissue was collected for the determination of histological and molecular biology.

The Animal Care and Use Committee of Guangxi Medical University approved all protocols.

### 4.2. Renal Morphology

After 10% neutral formaldehyde fixation, the renal tissue was dehydrated through a graded ethanol series and embedded in paraffin. Sections were prepared on a microtome and stained with Masson’s trichrome staining. Renal morphology was observed by light microscopy, the severity of the renal lesion was presented by the RIF index. Blue granular and linear deposits were interpreted as positive areas for collagen staining. Semi-quantitative evaluation was performed by computer-assisted image analysis (DMR + Q550, Leica Co., Germany). 400-fold original magnifications in ten fields (ignoring the fields containing glomerular parts) were measured, selected from coded sections for each rat at random. The extent of interstitial fibrosis was scored as: absent (0), involving less than 25% of the area (1), involving 26 to 50% of the area (2), and involving greater than 50% of the area (3) [29,30]. RIF index was obtained by the formula as follow: RIF index = (0 × *n*_0_ + 1 × *n*_1_ + 2 × *n*_2_ + 3 × *n*_3_)/(*n*_0_ + *n*_1_ + *n*_2_ + *n*_3_) = (0 × *n*_0_ + 1 × *n*_1_ + 2 × *n*_2_ + 3 × *n*_3_)/10; where *n*_0_, *n*_1_, *n*_2_ and *n*_3_ were the number of extent of interstitial fibrosis as absent, less than 25% of the area, 26 to 50% of the area, and greater than 50% of the area, respectively. All the fields were selected from coded sections for each rat at random and the scores obtained by two investigators were averaged.

### 4.3. Immunohistochemical Analysis of the Protein Expressions of Prohibitin, Transforming Growth Factor-β1 (TGF-β1), Collagen-IV (Col-IV), Fibronectin, α-Smooth Muscle Actin (α-SMA) and Cleaved Caspase-3

Kidney tissue fixed with 4% buffered paraformaldehyde was embedded in paraffin, and 4 μm thick sections were stained. The positive area was measured using a computer-aided manipulator (DMR + Q550, Leica Co., Germany). For immunohistochemical analysis of prohibitin, TGF-β1, Col-IV, fibronectin, α-SMA and cleaved Caspase-3, the sections were deparaffinized and washed with PBS. In order to retrieve the antigenicity from formalin fixation, the sections were incubated for 10 min in 10 mmol/L sodium citrate buffer using a microwave oven. After the incubation, all the sections were treated with 3% H_2_O_2_ in methanol for 10 min. All sections were then incubated with anti-prohibitin antibody (1:300) (Neomarker Lab, Co., USA), anti-TGF-β1 antibody (1:100) (Zhongshan, Co., Beijing, China), anti-Col-IV antibody (ready-to-use kit; Bo Shide, Co., Wuhan, China), anti-fibronectin antibody (1:50) (Zhongshan, Co., Beijing, China), anti-α-SMA antibody (ready-to-use kit; Shanghai Changdao, Co., Inc., China) and anti- cleaved- Caspase-3 antibody (1:200) (Thermo Fisher Scientific, Co., Runcorn, UK), respectively. After incubation with second antibody immunoglobulin (Shanghai Changdao, Co., Shanghai, China), the sections were stained with diaminobenizidine (Maixin Bio, Co., Fuzhou, China). Prohibitin, TGF-βl, Col-IV, fibronectin, α-SMA or cleaved Caspase-3 in renal tissue was measured by semi-quantitative evaluation. During evaluation of the interstitial areas, fields containing glomerular parts were ignored. All the evaluations were performed by two of the authors blind to the experimental code.

### 4.4. Apoptosis Assay

Cell apoptosis was examined by the TdT mediated dUTP nick end labelling (TUNEL) assay (Roche Inc., Basel, Switzerland) as described previously [31,32]. Six slides from each kidney were evaluated for percentage of apoptotic cells by using the TUNEL assay. Then 10 watch fields, which did not include the glomerular parts, were chosen at random under the microscope for each section. Brown staining of cell nuclei was on considered apoptotic cells. Positive brown cells and total cells were counted. The formula for the apoptosis index as the indicator of apoptosis was as follows [33,34]: cell apoptosis index = positive cells/total cells × 100%. The scores obtained by two investigators were averaged.

### 4.5. ROS Measurement

The cortical material of freshly dissected renal tissues was extracted, washed, weighed and homogenized in ice cold 0.9% normal saline solution. The homogenate was centrifuged at 4000 × g for 15 min at 25 °C and the supernatant was used for the determination [35]. The 500 μL of homogenates were used for detection of the ROS generation by the ROS sensitive dye, 2,7-dichlorodihydro-fluorescein diacetate (DCF-DA; Invitrogen, Co., USA), as an indicator [36,37]. Sample (50 μg protein) was incubated with 10 μL of DCF-DA (10 μM) for 3 h at 37 °C. The fluorescent product formed was measured by spectrofluorometer at 485/525 nm. Changes in fluorescence were expressed as an arbitrary unit [36].

### 4.6. Real Time Reverse Transcription Polymerase Chain Reaction to Detect Prohibitin/TGF-β1 mRNA Expression in Renal Tissue

Renal tissue was homogenized, and total RNA was extracted with TRIzol (Beijing Tiangen, Co., China). Primers were designed according to primer design principles by Primer Premier 5.0. The primers for prohibitin, TGF-βl and internal control β-actin were as follows: F 5′-TGGCGTTAGCGGTTACA GGAG- 3′ and R 5′-GAGGATGCGTAGTGTGATGTTGAC-3′ for prohibitin; F 5′-TGAGCACTGA AGCGAAAGCC-3′ and R 5′-GATTCAAGTCAACTGTGGAGCAAC-3′ for TGF-βl; F 5′-GCCCCT GAGGAGCACCCTGT-3′ and R 5′-ACGCTCGGTCAGGATCTTCA-3′ for β-actin. One microgram total RNA from the renal tissue of each rat was reverse transcribed into cDNA with an ExScript RT reagent kit (Takara Biotechnology, Co., Dalian, China). Prohibitin, TGF-βl and β-actin were amplified with SYBR Premix Ex Taq (Beijing Tiangen, Co., China). Gene expression of β-actin was also measured in each sample, and was used as an internal control for loading and reverse transcription efficiency. The analysis for each sample was performed in triplicate. The average threshold cycle (Ct, the cycles of template amplification to the threshold) was worked out as the value of each sample. The data of fold of changes was analyzed using 2^−ΔΔCt^ [38,39]. For example, the ΔΔCt of prohibitin mRNA expression in UUO group in 14-day was as follow: ΔΔCt_prohibitin,14-day,UUO group_ *=* (Ct_prohibitin, 14-day, UUO group_ − Ct_β-actin, 14-day, UUO group_) − (Ct_prohibitin, 14-day, SHO group_ − Ct_β-actin, 14-day, SHO group_), and the fold of changes of prohibitin mRNA expression in the UUO group in 14-days was 2^−ΔΔCt_prohibitin, 14-day, UUO group_^.

### 4.7. Statistical Analysis

The data are shown as mean ± standard deviation. Analysis of covariance and Student-Newman Keuls post-tests was performed to determine the differences among groups, and Pearson’s correlation coefficient was used to determine the relationships between the indicators. A value of *p* < 0.05 was considered as a significant difference. Statistical analysis was performed using the statistical package for social studies SPSS version 13.0 (SPSS, Chicago, IL, USA).

## 5. Conclusion

In conclusion, lower expression of prohibitin is associated with RIF progression, and ATRA treatment is associated with increased prohibitin in UUO rats, although the detailed mechanism is not fully understood. However, cell culture and further investigations should be conducted to explore the detailed mechanism.
