# Zhou, T.B., *et al.*, Correction: All-Trans Retinoic Acid Treatment Is Associated with Prohibitin Expression in Renal Interstitial Fibrosis Rats. *Int. J. Mol. Sci.* 2012, *13*, 2769–2782.

**DOI:** 10.3390/ijms131217295

**Published:** 2012-12-18

**Authors:** Tian-Biao Zhou, Yuan-Han Qin, Zheng-Yi Li, Hui-Ling Xu, Yan-Jun Zhao, Feng-Ying Lei

**Affiliations:** Department of Pediatrics, The First Affiliated Hospital of GuangXi Medical University, Nanning 530021, China; E-Mails: a126tianbiao@126.com (T.-B.Z.); zhengyili_gx@163.com (Z.-Y.L.); huilingxu01@163.com (H.-L.X.); yanjunzhao08@126.com (Y.-J.Z.); leifengyinggx@yahoo.cn (F.-Y.L.)

The authors wish to change Figure 2 of the paper published in *IJMS*[1]. The positions of H_1_ and H_2_ in the previous article were reversed. These errors have been amended in an amended version of the manuscript, which is available from the International Journal of Molecular Sciences website. The authors and publisher apologize for the inconvenience.

The corrected version can be accessed at: http://www.mdpi.com/1422-0067/13/12/17295/s1.
